# Death of Baron Portal

**Published:** 1832-09

**Authors:** 


### OBITUARY.

### DEATH OF BARON PORTAL.

THE year 1832 has been fatal to science; for within a few months have died five or six of the most illustrious men that France could boast: CHAMPOLLION, CUVIER, REMUSAT, SAINT MARTIN, and now to this catalogue of losses must be added that of M. Le Baron PORTAL, late chief physician to their Majesties Louis XVIII. and Charles X., honorary president of the Academy of Medicine, Professor of Anatomy in the "College de France," and in the Museum of Natural History, Knight of the order of St. Michael and of the Legion of Honour, member of the Institute, and of most of the learned Societies of Europe.

M. Portal was born at Gaillac (in the department of Tarn,) on the 5th of January, 1742, and died at Paris on the 23d of July, 1832. He suffered, like BUFFON, with a calculous disease, for the relief of which his extreme age prevented the ordinary surgical means being employed, and which at last destroyed him. He was ninety-one years old, and this consideration should assuage the poignancy of our regret, and make his loss be felt less sensibly than that of the other philosophers who have so lately gone before him to the grave; for they were almost all taken off in the flower of their age, and in the midst of their most important labours. With them have perished both the discoveries they would have made and the powerful influence of their presiding spirits: but M. Portal, almost a centegenarian, had long since terminated his scientific career; for the life of science lasts not so long as that of corporeal existence. He had already become an historical personage, though living, and some of our contemporaries will possibly feel surprised to hear the death of a physician announced, who was a professor of anatomy in the reign of Louis XV. The few lines that we consecrate to his memory may seem as if they had been written an age ago; but his life, which was so long, was devoted to science, and to medicine in particular; and it is our duty to recall to the remembrance of this forgetful generation a name which has so long since been nearly withdrawn from the more active scenes of life.

M. Portal, born in the southern provinces, commenced his medical education in the Montpelier School, then, next to Paris, the most celebrated in the kingdom. He soon distinguished himself by his unwearied industry and his passion for anatomical inquiries, at that time so much neglected. In 1766 he came to Paris, and devoted himself with renewed zeal to his favorite pursuit, anatomy. He was then twenty-four years of age. In 1768 he succeeded Ferrein in the chair of Anatomy in the "College de France;" and about the same time he was admitted a member of the Academy of Sciences. In 1777 he became professor of anatomy in the "Jardin des Plantes," and it was to the friendship of Buffon that he owed this situation, now vacant. Subsequently he devoted his attention to the practice of physic, and soon obtained one of those extensive practices which talent alone will not ensure, but to command which, to science must be added that unknown agent called fortune, or good luck.

It is probable to the extension of his practice that the change in the direction of his inquiries should be attributed. During the most laborious periods of his youth, he had abandoned himself almost wholly to anatomy and operative surgery. His earliest works related especially to surgical diseases, as will be seen on reference to his papers presented to the "Academie de Chirurgie." These essays contained novel views respecting the induration of the coats of the bladder occurring in old age, and on the mechanical treatment of dislocations; subjects essentially surgical. At this period surgery, which had been so long neglected, shone forth with unprecedented splendor, (thanks to the labours of the illustrious member of the academy founded by Mareschal.) Physic, but feebly supported in the schools of Paris, yielded the palm to her younger rival: and M. Portal, previously enlisted by choice in the service of anatomy, with facility turned his attention to surgery. His inquiries into pathological anatomy were primarily devoted to surgical diseases; and all the works that he published during the first fifteen years of his stay in Paris, from 1766 to 1780, were exclusively surgical.

M. Portal always maintained the most friendly relations with other members of his profession, and no one was more zealous in the performance of academic duties. Officially obliged to be present at all scientific ceremonies, he never shrunk from the fatigues of duty. Arrived at the age of eighty, when most men are infirm, even if alive, he found himself sufficiently energetic to accept the office of chief physician to his Majesty Louis XVIII.; and when Louis was dead, he was intrusted with the care of the health of Charles X., and it was with difficulty he was pursuaded not to undertake a similar charge for Louis-Philippe. At his death he was still in possession of two chairs, that he had occupied for upwards of fifty years. To the last he attended regularly to his practice, and never excused himself from a consultation.

The Baron Portal, like several other celebrated scientific men, was more known abroad than in France. His works are more frequently quoted by the English, the Italians, and the Germans, than by the French. To his more intimate acquaintances will belong the duty of furnishing biographical notices of his private life: we speak only of him as a public man, and we have been witnesses of the anxious respect which every where met him. Young and old made their obeisance to him, as to the memory of an age that had past. It is not for us to say whether these exterior signs of deference were homage rendered to dignity of character and the authority of science, rather than to the grey locks and venerable front of age. (*Gazette Medicale.*)

